# Solifenacin in Multiple Sclerosis Patients with Overactive Bladder: A Prospective Study

**DOI:** 10.1155/2011/834753

**Published:** 2011-05-05

**Authors:** Farida van Rey, John Heesakkers

**Affiliations:** Department of Urology, The Radboud University Nijmegen Medical Centre, Geert Grooteplein 10, 6500 GA Nijmegen, The Netherlands

## Abstract

*Objective*. To assess the efficacy and the effect on Qol of solifenacin for the treatment of OAB in MS patients. *Patients and Methods*. Thirty MS patients suffering from OAB were treated with solifenacin 5/10 mg for 8 weeks. The first 4 weeks patients received solifenacin 5 mg. At week 4 patients could request a dose increase to 10 mg. The efficacy was evaluated at 8 weeks. *Results*. After 4 weeks of treatment, 28 patients reported acceptable or no side effects. 17 continued the study with the 10 mg dosage, and 11 stayed on 5 mg solifenacin. Two patients withdrew from the study due to side effects. Solifenacin 5/10 mg for 8 weeks resulted in a significant decrease in number of micturitions and number of pads used per day compared to baseline. Also the severity of urgency prior to voiding decreased significantly, and an increase was seen in the volume per void. Twenty out of 30 patients chose to continue solifenacin therapy after termination of the study. The majority of patients reported global QoL improvement. *Conclusions*. Solifenacin is effective in the treatment of MS patients with OAB symptoms. This is the first study with solifenacin in a specific neurogenic patient group with a neurogenic disease-specific QoL outcome measure (MS-QoL 54).

## 1. Introduction

Solifenacin is a once-daily oral antimuscarinic agent that has been available in The Netherlands since September 2004 for the treatment of overactive bladder (OAB). The efficacy and safety of solifenacin has already been demonstrated in randomised, double-blind placebo controlled studies [1, 2]. However, the efficacy of solifenacin in multiple sclerosis (MS) patients with symptoms of OAB is unclear, since underlying neurological disease was an exclusion criterion in previous clinical studies. The aim of the present study was to assess the efficacy of solifenacin in MS patients with symptoms of OAB. To our knowledge to date this is the first and only study in which the efficacy of solifenacin for symptoms of OAB is evaluated in a neurogenic patient population. 

## 2. Patients and Methods

This is a prospective, open-label study to assess the efficacy and effect on quality of life of solifenacin 5/10 mg for 8 weeks in the treatment of MS patients with symptoms of OAB. The study protocol was approved by the local ethics committee of the University Medical Centre Nijmegen (CMO no. 2004/194). Patients provided written informed consent before enrolment. 

Men and women with a classified MS diagnosis according to the criteria of McDonald et al. [3] and symptoms of OAB were eligible for screening and study enrolment. Inclusion and exclusion criteria are listed in Table 1. None of the patients experienced a clinical relapse of their MS within 3 months prior to inclusion. Patients were evaluated at the outpatient clinic for symptoms of OAB by history, uroflowmetry, and determination of residual urine. Most patients underwent a urodynamic workup, but this was not mandatory for inclusion in the protocol. 

All medication that could influence bladder function was stopped at least 2 weeks prior to treatment or continued with no dose changes during the study. Patients were not allowed to use other antimuscarinic drugs prescribed for bladder dysfunction 2 weeks prior to or during the study. All methods, units, and definitions used in this study were done according to ICS standards [4].

At baseline patients were evaluated by 72-hour voiding diaries, as well as an MS-specific quality of life questionnaire (MS-QoL 54). The MS-QoL 54 is a multidimensional health-related quality of life measure that combines both generic and MS-specific items to a single instrument [5]. 

In the voiding diary the voiding frequency, voided volume per void, severity (degree) of urgency prior to any void, number of incontinence periods, severity of incontinence periods, and number of pads used were recorded. The degree of urgency was described on a scale of 0–3 (i.e., 0, no urge to void; 1, moderate urge to void; 2, normal urge to void that can still be suppressed; 3, severe urge to void that cannot be suppressed). The severity of urine loss was described on a scale of 0–3 (i.e., 0, no urine loss; 1, loss of some drops; 2, loss of a small amount; 3; severe loss possibly leading to a change of clothes).

Patients that met all inclusion criteria, and none of the exclusion criteria received solifenacin 5/10 mg for 8 weeks. All patients received solifenacin 5 mg once daily in the the first 4 weeks. At week 4, patients could continue 5 mg solifenacin treatment for another 4 weeks or request a dose escalation to 10 mg once daily in case of subjective insufficient efficacy and no or acceptable side effects. 

Both solifenacin 5 and 10 mg once daily have been shown to be effective and well tolerated for treating symptomatic OAB [6]. A starting dose of 5 mg solifenacin was chosen because of the somewhat favourable efficacy/side-effect ratio compared to 10 mg solifenacin.

After a total treatment period of 8 weeks the efficacy of solifenacin was evaluated by 72-hour voiding diary and MS-QoL 54. Global patient perception was assessed by enquiring patient satisfaction and the patients' desire to continue the treatment. 

Primary endpoints were defined as change from baseline in mean number of micturition per 24 hours, change from baseline in mean voided volume per void, and change from baseline in number of incontinence episodes per 24 hours and number of pads used per 24 hours. Change in quality of life (QoL), as measured with the MS-QoL 54, was a secondary endpoint.

Changes from baseline to endpoint were subjected to the Wilcoxon signed-ranks test.

The quality of life questionnaires were compared using the test for paired samples correlations (paired *t*-test).

## 3. Results

Between January and July 2005, 30 patients with MS and OAB symptoms were enrolled in this clinical study. All patients (12 men and 18 women) were diagnosed with OAB. Nine patients suffered from “OAB-dry” and 21 of “OAB-wet” Seven patients had received antimuscarinic therapy in the past without the desired effect. Twenty-three patients have not been treated with antimuscarinic agents prior to inclusion in the protocol. No patients had received other therapy than oral antimuscarinics for the OAB symptoms prior to the study.

After 4 weeks of treatment with solifenacin 5 mg, 28 patients reported acceptable or no side effects. Two patients withdrew from the study due to adverse events (gastrointestinal complaints and skin rash.) Both adverse events fully disappeared within days from discontinuation of the therapy. 

Of the remaining 28 patients, 11 patients (39%) chose to continue treatment with the 5 mg dosage. Seventeen (61%) patients requested a dose escalation to 10 mg solifenacin due to subjective insufficient efficacy. 

Evaluation of the voiding diaries after 8 weeks of treatment compared to baseline showed a significant decrease in median number of micturitions (−2.2 episodes/24 hours, *P* < .0001) and number of pads used per 24 hours (−1,0 pads/24 hours, *P* = .010). Also the degree of urgency prior to voiding decreased significantly (−12.6, *P* < .0001). Additionally a significant increase with 33 mL (*P* < .0001) was seen in the median voided volume per void. Whilst the median number of incontinence episodes per 24 hours and the median severity of urine loss improved numerically, these changes were not statistically significant (Table 2). This could be due to the low number of patients that were incontinent at study baseline.

Although many patients thus reported a subjective improvement in QoL, this was not apparent when analysing the MS-QoL 54. Only one of the subscales, health perception, showed a borderline significant increase of 0.8 points (*P* = .041). All other MS-QoL 54 subscales detected no significant differences from baseline to end of study (Table 3).

Global patient perception evaluation showed that 22 out of 30 patients (73%) chose to continue solifenacin therapy after the study, due to beneficial effects. Six patients (20%) chose to terminate the use of the drug because of lack of effect. Only in 2 out of 30 (7%) patients the reason for discontinuation of therapy was side effects.

## 4. Discussion

Lower urinary tract symptoms are common in MS. Up to 90% of patients have voiding complaints at some time during the course of the disease, especially with a disease duration longer than 10 years [7]. The most common voiding complaints are urgency, occurring in 24–86%, and urge incontinence, reported in 34–72% of patients [8].

Unlike the bladder dysfunction that follows spinal cord injury and causes life-threatening upper urinary tract conditions, MS very rarely causes severe upper urinary tract involvement but rather results in morbidity that influences the quality of life [9].

Anticholinergic agents are commonly used in the management of the OAB. For more than 30 years oxybutynin has been the drug of choice in patients with a neurogenic cause of OAB. Randomised controlled studies comparing oxybutynin to other antimuscarinic agents in neurogenic patients date from the late 1980s and early 1990s [10, 11]. A lot of neurogenic patients need a higher dose to achieve clinical efficacy [12]. With the introduction of tolterodine in 1999 a new antimuscarinic agent was added to the management options op OAB. Tolterodine is said to be equally effective compared to oxybutynin, but with a better side-effect profile [13]. Ethans et al. published the first randomised, controlled study comparing these two agents in neurogenic patients. It consisted of merely 10 patients [14]. In 2004 solifenacin was introduced for the treatment of OAB symptoms. In all pivotal studies neurogenic causes of an overactive bladder were considered an exclusion criterion [6, 15]. Therefore the efficacy of solifenacin in patients suffering from lower urinary tract dysfunction due to MS (or any other neurogenic cause) is unclear. However, OAB symptoms are very common in MS patients, and antimuscarinics are frequently prescribed, although the evidence for this indication is low. Therefore, even clinical studies with a relatively simple design are valuable in a specific group like MS patients.

Most of the time neurogenic patients are excluded from registration studies because they respond differently to medical treatments or they deal differently with common primary endpoints in OAB studies such as micturition frequency. When a wheelchair-bound patient has to tell you how many times he or she goes to the toilet per day, the answer will be highly dependent, amongst others, on environmental factors such as the availability of proper wheelchair toilets. Moreover MS is a progressive disease, so the clinical endpoint may differ from baseline to end of study even without intervention. The issue is that we do not have objective parameters to define a stable patient, apart from that MS patients are not a homogeneous group and that they may have all kinds of disabilities. 

This study shows that solifenacin is effective in the treatment of MS patients with OAB symptoms when assessed by means of 72-hour voiding diaries. Significant improvements were observed on severity of urgency, frequency and urge incontinence and number of pads used in 24 hours. Also subjective improvements evaluated by means of the global patient perception question suggested that patients were satisfied with the efficacy of solifenacin on their symptoms. 73% of patients wanted to continue therapy due to favourable results. We recognize that the study design is limited by the absence of a control group. It would be useful to do a head to head comparison in a randomized controlled trial in the future.

The effects on QoL, measured with the MS-QoL 54, were not apparent. The developers of the MS-QoL 54 utilized the Short Form 36 (SF-36) as the generic component to which 18 items were added to tap MS-specific issues [5]. It contains 54 questions distributed among 12 subscales (with 2 summary scores) and 2 single-item measures. The MS-QoL 54 contains only one question concerning bladder and/or bowel function (no. 51) and there is a subscale that specifically measures urological function/perception. Question 51 of the MS-QoL 54 informs about the degree in which patient were limited in their social activities during the last 4 weeks as result of their bladder and/or bowel dysfunction. We hypothesize that for this reason the MS-QoL 54 may be not sensitive enough to detect changes in the QoL due to lower urinary tract functioning. The domains of physical health, physical role limitations, health, perception and sexual function do show a trend towards increased QoL. Since lower urinary tract symptoms cause a substantial decrease in quality of life also in MS patients we expected that the improvement in lower urinary tract symptoms would have caused improvements in other domains of the MS-QoL 54 as well [16]. Therefore we think that the study is too small to show this effect. Urological problems are of major importance in the lives of MS patients, so we suggest that this domain should be incorporated in an MS-specific quality of life measuring instrument.

## 5. Conclusion

Solifenacin is efficacious in treating OAB symptoms in MS patients. Solifenacin significantly improved frequency, severity of urgency, volume voided, and number of pads used per 24 hours. 

The MS-QoL 54 showed no significant changes, other than a modest improvement in one of the subscales. Possibly, MS-QoL 54 is not specific enough to detect changes in OAB symptoms in an MS patient population. Further randomised clinical studies with antimuscarinics in this specific patient group are warranted.

## Figures and Tables

**Table 1:** 

Inclusion criteria (patients were eligible if all of the following applied)	
(1) Classified MS diagnosis	
(2) Written informed consent has been obtained	
(3) Patients are willing and able to complete the micturition diary correctly	
(4) Complaints of OAB	
(a) Urgency/Frequency (micturition frequency > 8/day)	
(b) Urge incontinence (involuntary loss of urine after a sensation of urge)	
Exclusion criteria (patients would be excluded from participation if any of the following apply)	
(1) Significant postvoid residual volume (PVR >200 mL)	
(2) Evidence of a urinary tract infection, chronic inflammation such as interstitial cystitis, bladder stones, previous pelvic radiation therapy, or previous or current malignant disease of the pelvic organs	
(3) Uncontrolled narrow angle glaucoma, urinary or gastric retention, or any other medical condition which in the opinion of the investigator makes the use of anticholinergics contra-indicated	
(4) Non-drug treatment including electrostimulation therapy or start of a bladder training program during the 12 weeks prior to or during the study	
(5) Use of drugs intended to treat urinary incontinence	
(6) Known or suspected hypersensitivity to other anticholinergics or lactose	
(7) Any clinical significant condition, which in the opinion of the investigator makes the patient unsuitable for the trial	
(8) Pregnancy or the wish to become pregnant during the study	

**Table 2:** 

	Baseline (IQD) *n* = 30	After 8 weeks of treatment (IQD) *n* = 28	Wilcoxon signed ranks Test
Median frequency/day	11.7 (9.3–13.4)	9.5 (6.9–10.9)	*P* = .000
Median volume voided/void	121.9 (103.1–152.9)	155.3 (103.1–198.2)	*P* = .000
Median incontinence episodes/day	1.3 (0.0–2.7)	0.2 (0.0–1.7)	*P* = .360
Median no. pads used/day	2.0 (0.0–3.4)	1.0 (0.0–2.0)	*P* = .010
Median severity of urine loss (0–3; daily added score)	1.2 (0.0–5.0)	0.3 (0.0–2.8)	*P* = .053
Median degree of urgency prior to voiding (0–3; daily added score)	36.3 (28.8–47.3)	23.7 (18.0–31.0)	*P* = .000

**Table 3:** 

*N* = 28		Mean	Std. deviation	Std. error mean	*P* value
Physical health	T1	25.9	19.7	4.8	.191
	T2	27.4	22.9	5.6	

Role limitation (physical)	T1	25.0	35.4	8.6	.173
	T2	32.4	35.1	8.5	

Role limitation (emotional)	T1	68.6	39.9	9.7	.985
	T2	62.7	42.3	10.3	

Pain	T1	68.2	28.9	7.0	.963
	T2	82.3	23.4	5.7	

Emotional well-being	T1	77.6	20.2	4.9	.761
	T2	72.7	13.2	3.2	

Energy	T1	47.5	22.5	5.5	.740
	T2	47.3	18.0	4.4	

Health perception	T1	36.2	18.2	4.4	.041
	T2	37.4	15.6	3.8	

Social function	T1	54.9	12.9	3.1	.631
	T2	58.8	12.3	3.0	

Cognitive function	T1	66.5	26.9	6.5	.669
	T2	76.5	19.0	4.6	

Health distress	T1	67.1	21.2	5.1	.517
	T2	69.4	21.4	5.2	

Sexual function	T1	44.6	31.3	7.6	.203
	T2	51.0	32.1	7.8	

Change in health	T1	27.9	26.3	6.4	.409
	T2	36.8	28.1	6.8	

Sexual satisfaction	T1	35.3	36.5	8.9	.394
	T2	44.1	37.0	9.0	

Overall QoL	T1	58.2	13.2	3.2	.725
	T2	57.7	12.0	2.9	

MS QoL54 (physical)	T1	44.3	15.3	3.7	.446
	T2	48.4	15.4	3.7	

MS QoL54 (mental)	T1	68.8	20.6	5.0	.863
	T2	67.7	17.5	4.2	

MS QoL54 (total)	T1	113.1	33.9	8.2	.955
	T2	116.1	30.9	7.5	

## References

[1] Chapple CR, Araño P, Bosch JLHR, De Ridder D, Kramer AEJL, Ridder AM (2004). Solifenacin appears effective and well tolerated in patients with symptomatic idiopathic detrusor overactivity in a placebo- and tolterodine-controlled phase 2 dose-finding study. *British Journal of Urology International*.

[2] Chapple CR, Cardozo L, Steers WD, Govier FE (2006). Solifenacin significantly improves all symptoms of overactive bladder syndrome. *International Journal of Clinical Practice*.

[3] McDonald WI, Compston A, Edan G (2001). Recommended diagnostic criteria for multiple sclerosis: guidelines from the International Panel on the Diagnosis of Multiple Sclerosis. *Annals of Neurology*.

[4] Abrams P, Cardozo L, Fall M (2002). The standardisation of terminology of lower urinary tract function: report from the standardisation sub-committee of the international continence society. *Neurourology and Urodynamics*.

[5] Vickrey BG, Hays RD, Harooni R, Myers LW, Ellison GW (1995). A health-related quality of life measure for multiple sclerosis. *Quality of Life Research*.

[6] Chapple CR, Rechberger T, Al-Shukri S (2004). Randomized, double-blind placebo-and tolterodine-controlled trial of the once-daily antimuscarinic agent solifenacin in patients with symptomatic overactive bladder. *British Journal of Urology International*.

[7] McGuire EJ, Savastano JA (1984). Urodynamic findings and long-term outcome management of patients with multiple sclerosis-induced lower urinary tract dysfunction. *Journal of Urology*.

[8] Betts CD, D'Mellow MT, Fowler CJ (1993). Urinary symptoms and the neurological features of bladder dysfunction in multiple sclerosis. *Journal of Neurology Neurosurgery and Psychiatry*.

[9] Koldewijn EL, Hommes OR, Lemmens WAJG, Debruyne FMJ, Van Kerrebroeck PEV (1995). Relationship between lower urinary tract abnormalities and disease- related parameters in multiple sclerosis. *Journal of Urology*.

[10] Gajewski JB, Awad SA (1986). Oxybutynin versus propantheline in patients with multiple sclerosis and detrusor hyperreflexia. *Journal of Urology*.

[11] Thuroff JW, Bunke B, Ebner A (1991). Randomized, double-blind, multicenter trial on treatment of frequency, urgency and incontinence related to detrusor hyperactivity: oxybutynin versus propantheline versus placebo. *Journal of Urology*.

[12] Bennett N, O’Leary M, Patel AS, Xavier M, Erickson JR, Chancellor MB (2004). Can higher doses of oxybutynin improve efficacy in neurogenic bladder?. *Journal of Urology*.

[13] Serels SR, Appell RA (1999). Tolterodine: a new antimuscarinic agent for the treatment of the overactive bladder. *Expert Opinion on Investigational Drugs*.

[14] Ethans KD, Nance PW, Bard RJ, Casey AR, Schryvers OI (2004). Efficacy and safety of tolterodine in people with neurogenic detrusor overactivity. *Journal of Spinal Cord Medicine*.

[15] Cardozo L, Lisec M, Millard R (2004). Randomized, double-blind placebo controlled trial of the once daily antimuscarinic agent solifenacin succinate in patients with overactive bladder. *Journal of Urology*.

[16] Quarto G, Autorino R, Gallo A (2007). Quality of life in women with multiple sclerosis and overactive bladder syndrome. *International Urogynecology Journal and Pelvic Floor Dysfunction*.

